# Impact of co-fermenting modified camel milk using yogurt starter culture and *Limosilactobacillus fermentum* on the quality of quinoa-supplemented fermented milk products

**DOI:** 10.3389/fnut.2025.1703215

**Published:** 2025-12-05

**Authors:** Amjad F. A. Alsadun, Sally S. Sakr, Asmahan A. Ali, Khalid A. Alsaleem, Mohammed Aladhadh, Thamer Aljutaily, Mona S. Almujaydil, Raed Alayouni, Ateteallah Hassan, Hassan Barakat, Mohamed F. Y. Hassan

**Affiliations:** 1 Department of Food Science and Human Nutrition, College of Agriculture and Food, Qassim University, Buraydah, Saudi Arabia; 2 Department of Dairy Science, Faculty of Agriculture, Sohag University, Sohag, Egypt

**Keywords:** camel milk, fermentation, quinoa, whey protein isolate, *Limosilactobacillus fermentum*, microstructure, antioxidant activity, food supply

## Abstract

**Introduction:**

Fermented camel milk represents a functional dairy product of significant nutritional and pro-biotic value, yet optimization of its coagulation properties and bioactive compound content remains under-studied.

**Methods:**

This study investigated the enhancement of coagulation quality in fermented camel milk by in-corporating whey protein isolate (WPI) and trisodium citrate (TSC) to facilitate improved acid coagulation. Additionally, the research examines the effects of quinoa seed preparations on the quality of the resultant products. In the preliminary phase, camel milk was supplemented with either 1.5% or 2% WPI in conjunction with TSC at a concentration of 30 mmol L^−1^, analyzing the resultant fermentation characteristics.

**Results:**

Camel milk with 2% WPI and TSC significantly expedited acidification and reduced fermentation time. Furthermore, this formulation enhanced gel formation and viscosity during fermentation with the yoghurt starter culture (YC–381) and *Limosilactobacillus fermentum* (B–1932). Due to these promising outcomes, this formulation was chosen for the subsequent study phase. In the second phase, camel milk that had undergone pre-modification before fermentation received 1–3% additions of quinoa sprout flour (QSF) or freeze-dried aqueous quinoa extract (AQE). The blended fermented products were assessed for their physicochemical, microbiological, and antioxidant properties, as well as sensory and microstructural characteristics over a 15-day storage period at 4 ± 1 °C. Notably, all quinoa preparations, particularly the 3% AQE supplementation, sig-nificantly accelerated acidification, improved water-holding capacity, and increased antioxidant activity in the blended samples. The microstructural analysis revealed that samples containing 3% AQE developed a gel network characterized by reduced pore size and enhanced porosity. In addition, incorporating AQE facilitated greater viability of starter cultures compared to QSF, likely attributed to its superior nutrient availability.

**Discussion:**

The co-fermentation of modified camel milk with a yoghurt starter culture and *L. fermentum*, in combination with quinoa supplementation, enhanced fermentation efficiency, functional properties, and pro-biotic viability.

## 1 Introduction

The total population of camels is 35 million and 492,853 head in the world and in Saudi Arabia, respectively (1). The dromedary camel (*Camelus dromedarius*) is considered an essential animal and the primary meat and milk source in Saudi Arabia (2). Nowadays, dairy products from camel milk (CM) attract consumers not only in the Gulf but also in European markets (3). Fresh and fermented CM is vital in dry regions where camels are kept for food and other purposes. It is eaten and used to treat several diseases (4). A modest quantity of lactose and cholesterol makes CM like human milk but different from other animal milks. It also contains many minerals, including magnesium, sodium, iron, potassium, zinc, and copper (5, 6).

Furthermore, it has served as a vital source of sustenance for nomadic and pastoral societies in arid locations globally for generations. Recently, cow’s milk alternatives, particularly CM, have gained popularity owing to their superior nutritional value and medicinal benefits (7). It is a natural health product due to its anti-inflammatory, immunomodulatory, antioxidant, insulin-mimetic, and anti-apoptotic characteristics. Furthermore, it comprises reactive proteins that could enhance immune protection and have antiviral and antibacterial activities (8). Bioactive peptides produced from CM via enzymatic hydrolysis or fermentation have recently garnered attention. Much research has examined the health benefits of these peptides, either from whole milk or proteins like whey or casein (9, 10). Consequently, the dairy sector may allow CM and its products to expand (11).

Unfortunately, CM is challenging to process into commercially viable dairy products due to consumer acceptability and manufacturing issues (12). CM coagulates slowly and produces weak, poorly structured products. The difference in behavior between CM and bovine milk may be attributed to the presence of gigantic casein micelles, casein fraction distribution, and the absence of *β*-lactoglobulin (β-Lg). Fermented CM’s weak structure may also be due to its tiny fat globule (3, 13). Despite these obstacles, investigations show that altering the bovine milk production procedure can help fermented CM products. Recent studies have attempted fermented CM with mixed results (14, 15). Thus, bovine skimmed milk powder, whey protein products, microbial transglutaminase, hydrocolloids, and stabilizers have been used to ferment CM (3). Another promising approach is the addition of calcium chelators (11). Many researchers have found that calcium-chelating compounds can break down native casein micelles into smaller particles, improving acid gel characteristics. Adding calcium-chelating chemicals (trisodium citrate, sodium phosphate, etc.) to micellar casein dispersions dissociates native casein micelles into smaller aggregates (11, 16). Previous research has shown that commercial yogurt starter cultures, used to ferment cow and goat milk, ferment CM more slowly (17). This could be attributed to differences in proteolysis between the two types of milk rather than the presence of inhibitory substances in CM (14). Recently, Seifu (13) recommended using *L. fermentum* as a co-culture due to its high proteolytic activity and ability to improve fermented CM’s sensory quality.

In many countries, legumes (lentils, peas, chickpeas, beans, etc.) and grains like quinoa and their constituents are being used in functional food formulations, and their usage as fortifiers could enhance their consumption, satisfying strongly suggested dietary standards (18–20). Quinoa flour in yogurt, a fermented dairy product, can improve its nutritional value (21–23) and quality (18). Previously, Alkobeisi et al. (24) tested quinoa flour as a skim milk powder substitute in concentrated low-fat yogurt. Replacing skim milk powder with quinoa flour changed yogurt’s gel hardness, syneresis, and viscosity. They found that adding more quinoa flour improved yogurt texture and viscosity and sped up fermentation compared to free quinoa flour. Earlier, the study by El-shafei et al. (25) revealed that partially replacing goat’s milk with liquid quinoa extract improved the texture, microstructure, and taste of the yogurt. It also enhanced the viability of lactic acid bacteria and reduced fermentation time. Quinoa sprout powder and extract are generally recognized as safe (GRAS) (26) and can be used as a nutrient source. Researchers are studying quinoa sprouting to increase its functional, technical, and nutritional qualities, boosting its use in food compositions (27). Germination of quinoa improves nutrient content and reduces anti-nutritional factors like saponins (28, 29). Sprouting quinoa can be an economical and straightforward bio-process for expanding food product variety, potentially improving specific consumer traits (27, 30).

This study aims to examine the possibility of enhancing the coagulation quality of fermented CM. The method entailed augmenting it with WPI and incorporating TSC to establish processing procedures applicable to CM, thus enhancing its suitability for acid coagulation. Furthermore, after analyzing the results from the initial phase of the study, the researchers assessed the impact of including quinoa seed preparations to enhance the quality and characteristics of fermented CM products.

## 2 Materials and methods

### 2.1 Materials

Raw CM (Total solids: 11.10 ± 0.02%, Protein: 3.24 ± 0.11%, Fat: 4.36 ± 0.04%, Ash: 0.75 ± 0.12% and Lactose: 2.75 ± 0.21%) was freshly collected during the morning milking of Wadah species (aged from 5 to 8 years) from local breeders in the Qassim region, Saudi Arabia, immediately after milking was kept under cooling conditions (4 ± 1 *°*C) and directly transferred to the lab (Food Science and Human Nutrition Department-girls section, College of Agriculture and Food, Qassim University, Saudi Arabia) for further preparations. WPI (~93% protein) was purchased online through the i-herb website (ALLMAX IsoNatural, no artificial colors, flavors, or added sugars). White Quinoa seeds (*Chenopodium quinoa*, 7.1% fat, 12.3% protein, 67.9% carbohydrates, and 7.1% dietary fibers) were obtained from a local Qassim region, Saudi Arabia store. Direct Vat Set (DVS) YC-381 commercial starter culture (Chr. Hansen Laboratories, Copenhagen, Denmark) containing *Lactobacillus delbrueckii* subsp. *bulgaricus* and *Streptococcus thermophilus* w*ere* purchased from Misr Food Additives (MIFAD, Badr City, Egypt). *L. fermentum* (B-1932) strain was procured as a gift from the Agriculture Research Service (ARS) Culture Collection, Norwegian Radio Relay League (NRRL), Peoria, USA. De Man, Rogosa, and Sharpe (MRS) broth and agar media, M17 agar, MacConkey agar, Malt extract agar, and Nutrient agar were purchased from Condalab, Calle Forja 9, 28850, Torrejón de Ardoz, Madrid, Spain. All chemicals used in the study were of analytical grade. Chemicals for each experimental method are detailed under its method of analysis and described subsequently.

### 2.2 Preparation methods

#### 2.2.1 Preparation of active *L. fermentum* (B-1932) starter culture

The *L. fermentum* (B-1932) strain was activated in MRS broth at 37 °C overnight for five-day intervals, followed by a sixth passage in sterilized reconstituted skim milk (9% TS) containing 1% w/v glucose at 37 °C overnight to prepare a starter culture available to be used in the rest of the study (31).

#### 2.2.2 Preparation of quinoa sprouts (QS) flour

Quinoa seeds were sprouted according to the instructions reported by Suárez-Estrella et al. (30) and Enciso-Roca et al. (32), with some modifications. Briefly, whole clean quinoa seeds (1 kg) were soaked in water (1:1, w/w) for 24 h at room temperature, and water was replaced every 12 h. After removing excessive moisture, the wet seeds were moistened on muslin with distilled water and incubated at refrigeration temperature (between 4 and 6 °C) for 48 h. The seeds were washed every 6 h and then re-refrigerated until good sprouts had been obtained. Sprouted seeds were harvested and dried (55 °C for 6 h) in a forced convection stove to stop enzyme activity. Finally, the dried sprouted seeds were ground in a small mill (Thomas Wiley, Philadelphia, PA, USA) at speed 6 for 2 min, double sieved (500 μm), and stored at −20 °C until use.

#### 2.2.3 Preparation of freeze-dried aqueous quinoa extract (QE)

According to El-Shafei et al. (25), the aqueous Quinoa extract was prepared. Clean seeds were first soaked for 8 h in water before washing them several times with running water. Then, the seeds were drained and blended with warm water (50 °C) at a ratio of 1:1 w/w before grinding for about 5 min. For aqueous quinoa extract harvesting, the mixed seeds were filtered through a double-layer muslin, and the filtrate was freeze-dried (CHRIST, Alpha 1–2 L.D. plus, Germany) for 96 h at −48 *°*C under a pressure of 0.032 mbar.

#### 2.2.4 Preparation of fermented CM samples

To optimize the CM fermentation conditions, the suitable WPI amount to improve the fermentation quality of CM was first determined. A co-fermentation with traditional yogurt starter culture (YC-381: 0.2gL^−1^) and *L. fermentum* (B-1932: 2%) was used as a first step in the current study to evaluate the progression rate of the acidity during the fermentation process, and the changes in apparent viscosity in the fermented came milk (FCM) final products prepared from CM modified by TSC (30 mmol L^−1^) with different concentrations of WPI (1.5 and 2%). In this step, four treatments of fresh CM before co-fermentation by yogurt starter culture (YC-381) and *L. fermentum* (B-1932) were applied. The changes in pH values and percentage of titrable acidity (TA%) during fermentation for T1 (CM supplemented with 1.5% WPI), T2 (CM supplemented with 1.5% WPI and 30 mm L^−1^ TSC), T3 (CM supplemented with 2% WPI), and T4 (CM supplemented with 2% WPI and 30 mmol L^−1^ TSC) CM samples were monitored during incubation at 40 °C. The apparent viscosity of fermented samples was measured after 1 day of cold storage.

**Figure 1:**
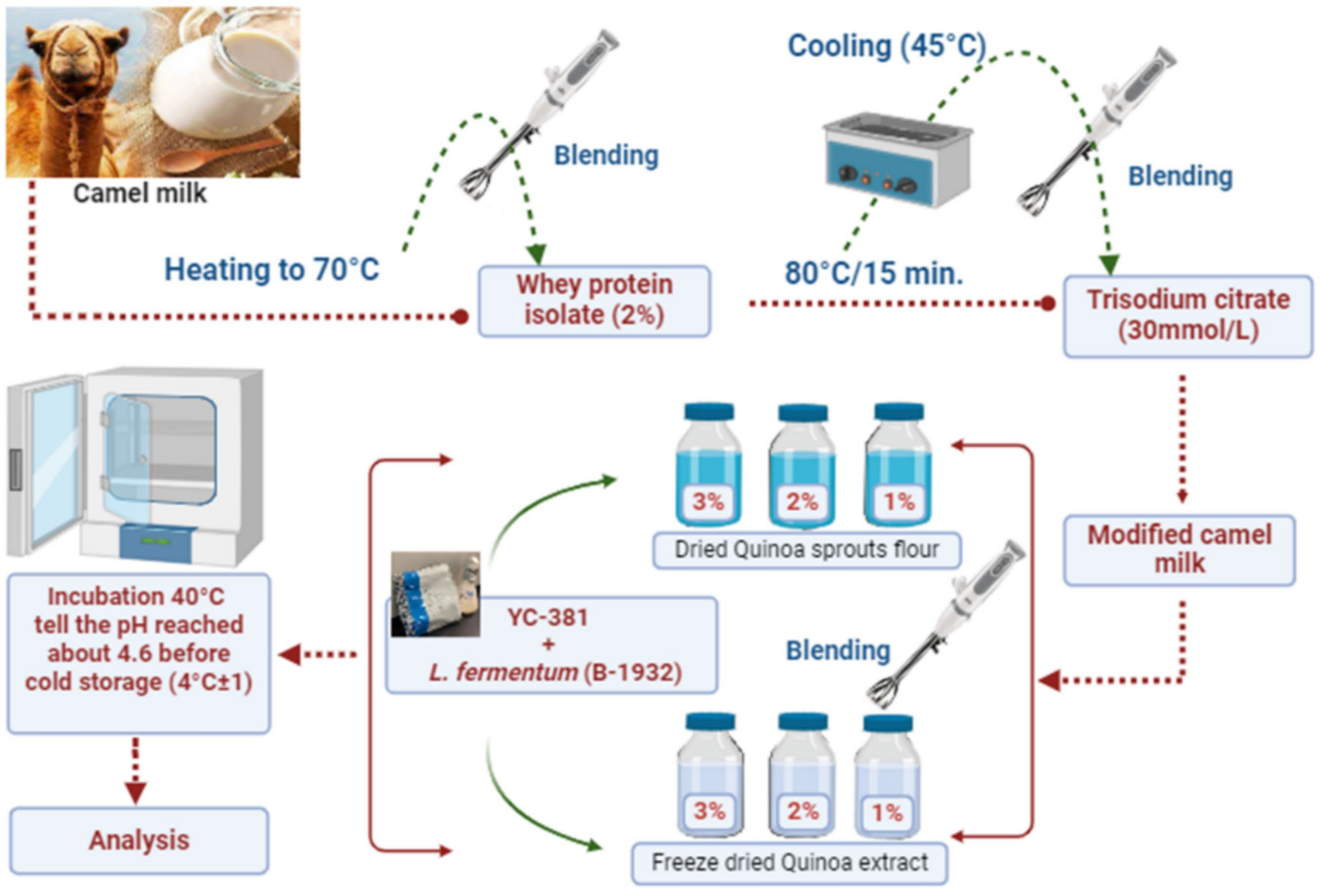
Summary of the fermented CM samples preparation method.

Then, seven fermented CM samples were prepared, as described in Figure 1. First, 14 L of fresh CM for each batch was preheated in a water bath to about 70 °C, and WPI (2%) was added under continuous blending. The milk was then heat treated (80 °C 15 min^−1^) and cooled to about 45 °C before the extra pure (98%) tri-sodium citrate dihydrate (TSC, Loba Chemie Pvt. Ltd., Mumbai, India) addition (30 mmol L^−1^). Then, the modified heat-treated CM was separated into seven portions. The first portion (FCM-C) was a control (without Quinoa incorporation). Dried Quinoa sprout flour was added to the three milk portions at concentrations of 1% (FCM-QS1), 2% (FCM-QS2), and 3% (FCM-QS3).

The remaining three portions were supplemented with 1% (FCM-QE1), 2% (FCM-QE2), and 3% (FCM-QE3) of freeze-dried aqueous Quinoa extract. All milk samples were subjected to fermentation by yogurt starter culture (YC-381) at the level of 0.2 g L^−1^ along with *L. fermentum* (B-1932: ARS Culture Collection, USA) at the level of 2% at 40 °C until the pH reached about 4.6, then kept refrigerated for analysis. The whole experiment was carried out in triplicate.

### 2.3 Methods of analysis

#### 2.3.1 Chemical composition

The total protein, total solids (TS), and ash contents were determined according to the methods described in the Association of Official Analytical Chemists methods (33). The fat content was determined by the micro-Folch extraction method (34). The amount of total carbohydrates was calculated using the following equation:


(1) Total carbohydrates%=Total solids−(Fat+Protein+Ash)

#### 2.3.2 Titratable acidity (TA%) and pH

The titratable acidity of FCM (expressed as lactic acid %) was determined by titration with 0.1 N NaOH using phenolphthalein as an indicator (33). The pH of different samples was measured fresh, during fermentation, and at the end of fermentation using a digital pH meter (HANNA HI 8314 Portable).

#### 2.3.3 Antioxidant activity assay

The 2,2-diphenyl-1-picrylhydrazyl (DPPH) radical scavenging activity of the samples was determined using a modified protocol based on the method described by Mudgil et al. (35). The absorbance at 517 nm was measured using a 96-well microplate reader (Multiskan Sky, Thermo Fisher Scientific, Cambridge, MA, USA) after 30 min incubation at 37 °C. 25 μL of each soluble nitrogen extract was added to each microplate well and mixed with 275 μL of DPPH reagent (0.1 mmol L^−1^ in 95% methanol). The mixture was then left in the dark for 30 min before measurement. The radical scavenging activity % of extracts was calculated using the following formula:


(2) $$\text{DPPH radical scavenging activity}\;(\%)=[(A_{0}-A_{s})/A_{0}]\times 100$$

where A_0_ represents the absorbance at 517 nm of the blank (distilled water), and A_s_ is the absorbance at 517 nm of the extract. Furthermore, a standard curve equation using Trolox was utilized to calculate the μM Trolox equivalent gL^−1^ dry weight (DW).

#### 2.3.4 Phytochemical determination

The total phenolic content (TPC) values of the extract prepared from each sample were determined using the Folin–Ciocalteu reagent, as per the method outlined by Bettaieb et al. (36). In brief, 150 μL of the sample was mixed with 300 μL of Folin–Ciocalteu reagent in Eppendorf tubes and agitated for 5 min. Subsequently, 300 μL of an alkaline solution (7.5% sodium carbonate solution, Na_2_CO_3_) was added to the mixture to arrest the reaction. The mixture was then incubated in the dark for 60 min at 23 °C, centrifuged (Sigma, 3 K30, Germany) at 10,000×g for 10 min at 4 °C, and 120 μL of supernatant from each Eppendorf was transferred to a new plate. The absorbance was measured at 760 nm using a microplate reader (BioTek, Winooski, VT, USA), and the measurements were compared to the standard curve of gallic acid (GA) solution (R2 = 0.99). TPC content per gram of dry weight (DW) was expressed as milligrams of gallic acid equivalents (mg GAE g^−1^ DW). The total flavonoid (TF) content of FCM samples was determined according to the method of Mohdaly et al. (37). The TF content was presented as mg quercetin equivalent (QE) g^−1^ DW. The total flavonols (TFL) content was determined according to Kumaran and Karunakaran (38). The absorbance at 440 nm was measured, and then TFL content was expressed as mg quercetin equivalent (QE) g^−1^ DW.

#### 2.3.5 Microbiological assay

For detecting the microbiological quality of FCM samples during cold storage, coliform bacteria, total mold and yeast, and total bacterial counts were enumerated using MacConkey agar, malt extract agar, and Nutrient agar media, respectively, by counting colony-forming units (CFU) after plating serial dilutions according to Robertson et al. (39). The De Man, Rogosa, and Sharpe (MRS) medium detected *Lactobacillus* strains from dilutions 6 through 9. Two layers of medium were poured over diluted samples, left for solidification, and then incubated under anaerobic conditions at 37 °C for 48 h. The *Streptococcus thermophilus* was detected from the 6th to the 9th dilutions for each sample by enumeration on M17 agar medium, and the plates were aerobically incubated at 37 °C for 24 h.

#### 2.3.6 Apparent viscosity

The apparent viscosity (cp) of FCM samples was estimated in triplicate during 15 days of cold storage (4 ± 1 °C) using the Fungilab Rotational Viscometer. Each Fermented CM sample was adapted to about 10 °C, then poured into a glass beaker. RV spindle marking No. 4 was lowered perpendicularly into each beaker and rotated at 100 rpm. When stable values were obtained, viscosity measurements were recorded.

#### 2.3.7 Water holding capacity (WHC)

The WHC of the fermented milk was determined according to Akalin et al. (40). A sample of about 20 g of fermented milk (FM) was centrifuged at 5,000×*g* for 10 min at 20 °C (Sigma, 3K30, Germany). The whey expelled (WE) was removed and weighed in grams. The WHC was calculated as follows:


(3) WHC(%)=[(FM−WE)/FM]×100

#### 2.3.8 Instrumental color determination

According to the method described by Alqahtani et al. (41), the changes in the color of FCM samples during 15 days of cold storage were determined. The instrumental color determinations were performed using the Hunter Lab Minolta colorimeter with a 20 mm aperture set at illumination D65 and 100 standard observer angles. Before analyzing the measurements for color changes, the initial Hunter color values, including lightness (L*), redness (a*), and yellowness (b*), were calibrated against white and black standard discs. Each sample was carefully prepared by pouring 10 mL of the liquid into a petri dish, resulting in a liquid layer approximately 1 cm high. Three distinct readings at random positions on the sample surface were taken for each analyzed sample. The chroma (C), color changes (∆E), and browning index (BI) were then calculated according to Lavelli et al. (42) and compared with the values of the control.

#### 2.3.9 Scanning electron microscope (SEM)

Recio et al. (43) described a technique for the FCM sample’s microstructure fixation. Fresh FCM samples were examined by SEM (JEOL JEM-2100, USA), and micrographs were taken at 5,000× magnification. The average Pore diameter (μm) that appeared in the Scanning electron micrographs of different FCM samples was then calculated using ImageJ and presented by Origin 2023 software.

### 2.4 Statistical analysis

The statistical analysis was conducted using the SPSS program (v.19), which has a multi-function utility regarding the experimental design and multiple comparisons using two-way ANOVA. Differences were considered to be statistically significant at (*p*<0.05), according to Steel et al. (44).

## 3 Results

### 3.1 Effect of WPI and TSC on the fermentation characteristics of CM and the viscosity of FCM samples

**Figure 2:**
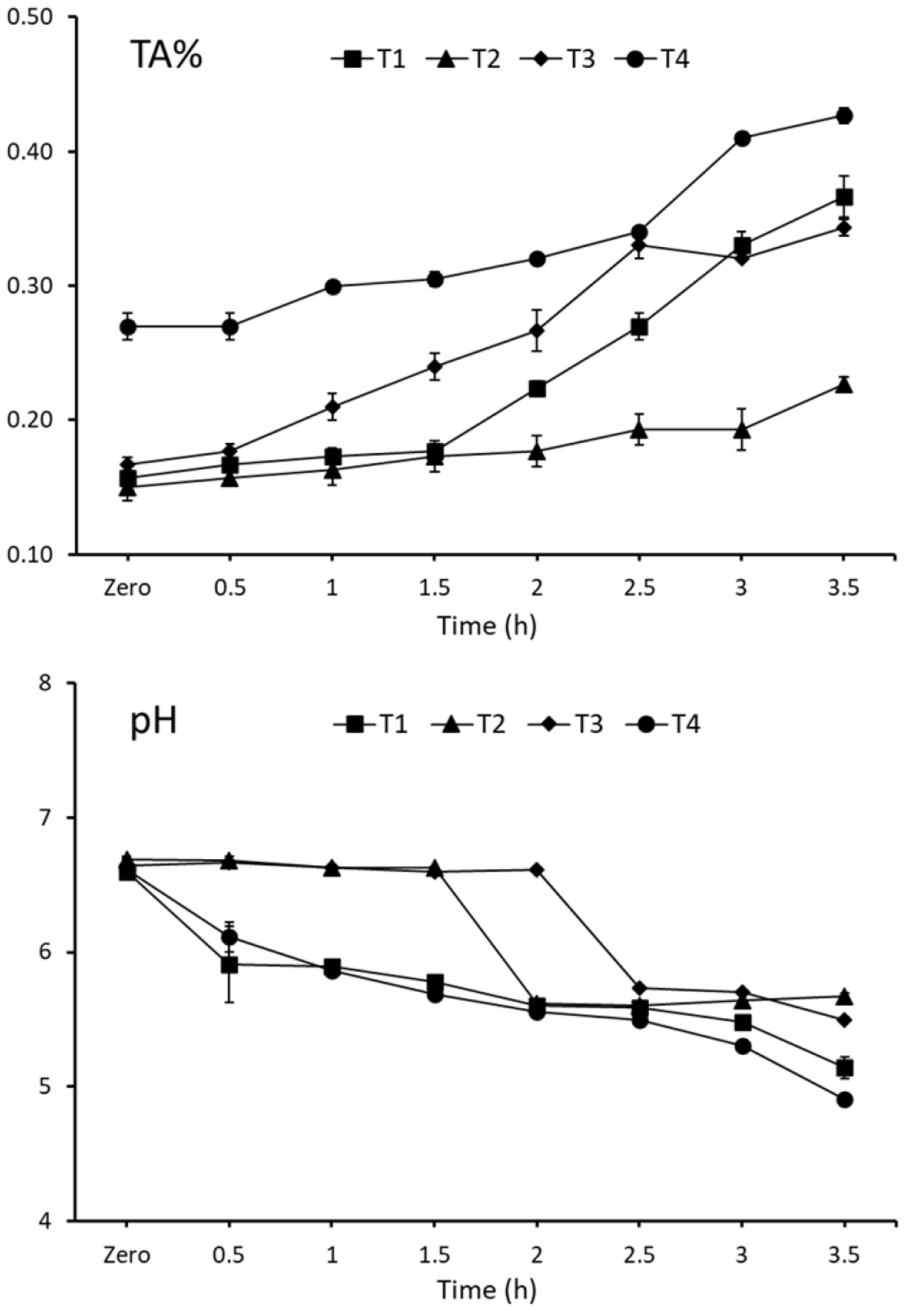
Changes in pH values and TA% during fermentation of CM as affected by the addition of WPI with or without TSC. T1: CM supplemented with 1.5% WPI, T2: CM supplemented with 1.5% WPI and 30 mmol L^−1^ TSC, T3: CM supplemented with 2% WPI, T4: CM supplemented with 2% WPI and 30 mmol L^−1^ TSC (mean ± SE), *n* = 3.

**Figure 3:**
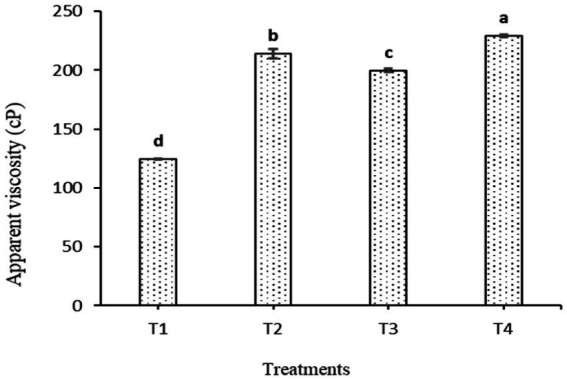
Changes in apparent viscosity (cp) of FCM after 1 day of cold storage (4 ± 1 °C) as affected by the addition of WPI with or without TSC. T1: CM supplemented with 1.5% WPI, T2: CM supplemented with 1.5% WPI and 30 mmol L^−1^ TSC, T3: CM supplemented with 2% WPI, T4: CM supplemented with 2% WPI and 30 mmol L^−1^ TSC (mean ± SE), *n* = 3.

Data from the first step of the study are presented in Figures 2, 3. The changes in pH values and titratable acidity% (TA%) in the CM modified by adding 2% WPI and 30 mmol L^−1^ TSC (T4) represented a more rapid decrease in pH and an increase in TA% during fermentation than other samples. By fermentation reaching 3.5 h, the pH and TA% were recorded at 4.9 and 0.43% for the T4 sample, respectively. While the pH values were approximately 5.1, 5.7, and 5.5, and the TA% was 0.37, 0.23, and 0.34% for T1, T2, and T3, respectively, after 3.5 h of fermentation at approximately 40 °C. There was a significant difference in TA% between all samples and during fermentation periods for each sample, as presented in Figure 2. The increase in TA% was slightly significant during the 1.5 h of fermentation in all four samples. The TA% was significantly higher (*p* ≤ 0.05) in T4 after approximately 3.5 h of fermentation than in the other three samples, T1, T2, and T3.

The apparent viscosity (cp) for all produced FCM samples determined after 1 day of cold storage is presented in Figure 3. Significant differences (*p* < 0.05) in apparent viscosity were observed between all FCM samples. Adding TSC significantly increased the viscosity of FCM samples (T2 and T4) compared to samples fortified with WPI and free of TSC (T1 and T3). The higher apparent viscosity (299.13 cp) was recorded for the T4 sample, which was fortified with 2% WPI and 30 mmol L^−1^ TSC, than all other samples, and the lowest (124.75 cp) was for the T1 sample, which was modified only by 1.5% WPI. From the obtained results, it could be noted that the co-fermentation of yogurt starter culture (YC-381) and *L. fermentum* (B-1392) of modified CM by the addition of WPI (2%) along with TSC (30 mmol L^−1^) could help accelerate acidity progression, improve acid coagulum, and increase milk viscosity. Thus, adding WPI (2%) along with TSC (30 mmol L^−1^) was chosen to modify CM before fermentation in the rest of the current study.

### 3.2 Changes in pH and TA% of FCM samples during 15 days of cold storage (4 °C ± 1)

**Figure 4:**
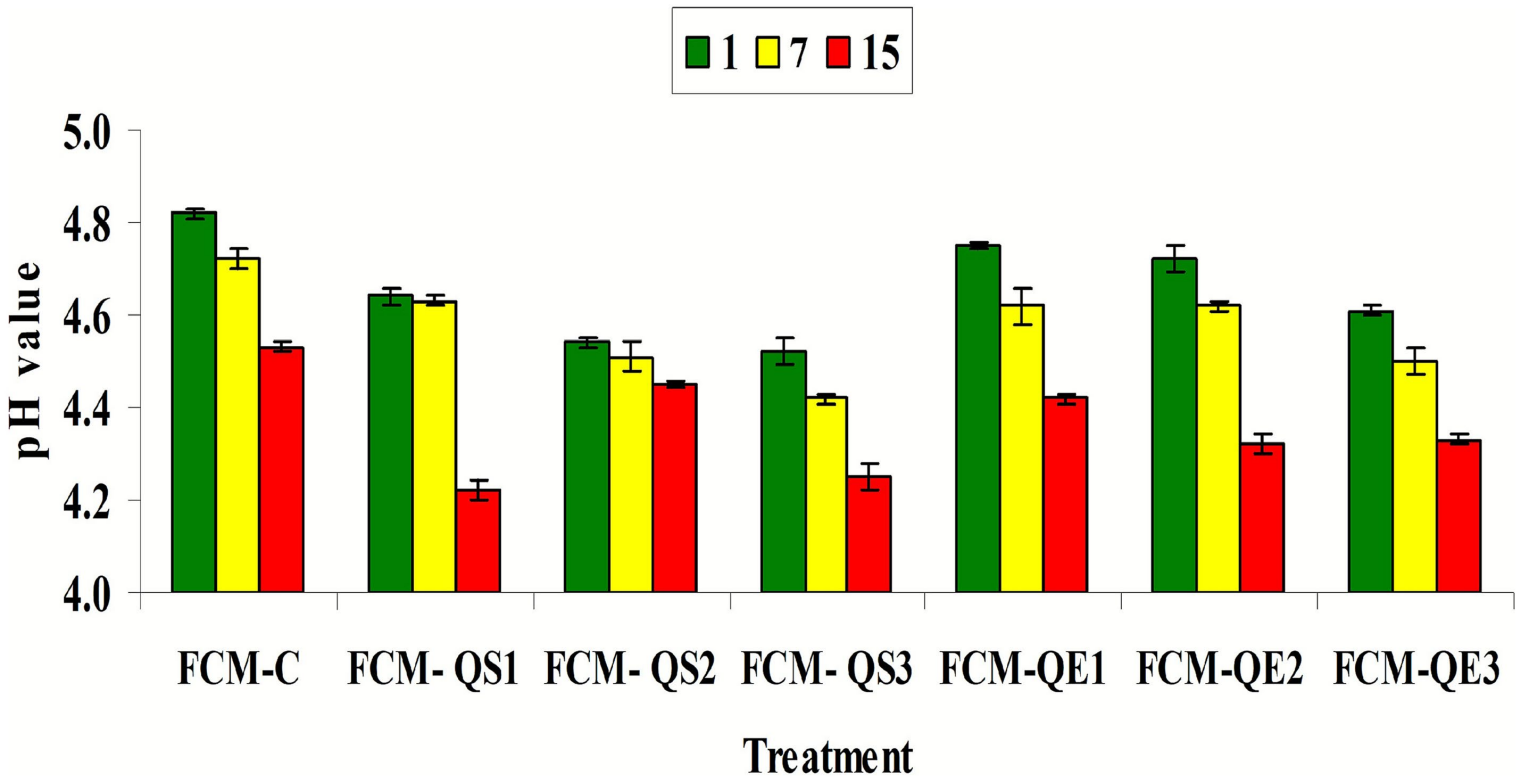
pH values of different FCM samples during 15 days of cold storage (4 °C ± 1) as affected by quinoa preparations addition. FCM-C (control without quinoa), FCM-QS1 (1% quinoa sprout flour), FCM-QS2 (2% quinoa sprout flour), FCM-QS3 (3% quinoa sprout flour), FCM-QE1 (1% freeze-dried aqueous Quinoa extract), FCM-QE2 (2% freeze-dried aqueous Quinoa extract), and FCM-QE3 (3% freeze-dried aqueous Quinoa extract).

Changes in pH values and TA% in different FCM samples during 15 days of cold storage (4 °C ± 1) as affected by quinoa preparations addition are presented in Figure 4 and Table 1. Regarding the pH values, it is clearly observed that fermented CM free from any quinoa preparations (FCM-C) had a higher pH than all other samples on all days of storage. Furthermore, supplementing CM with quinoa sprout flour led to a more effective decrease in pH values than freeze-dried aqueous quinoa extract at each storage day. Moreover, it was detected that the reduction in pH became higher due to increasing the quinoa addition and the storage period. This behavior can be directly related to the effect of Quinoa substances in accelerating the growth of the starter culture, causing more acidity.

**Table 1:** TA% of different FCM samples during 15 days of cold storage (4 °C ± 1) as affected by the addition of quinoa preparations addition.

Sample	Storage period (day)		
	1	7	15
FCM-C	0.86 ± 0.01^bB^	0.88 ± 0.00^bcAB^	0.89 ± 0.00c^A^
FCM-QS1	0.89 ± 0.01^bB^	0.90 ± 0.02^bAB^	0.92 ± 0.02^bcA^
FCM-QS2	0.90 ± 0.01^bB^	0.91 ± 0.01^bB^	0.95 ± 0.01^abA^
FCM-QS3	0.96 ± 0.01^aA^	0.96 ± 0.01^aA^	0.98 ± 0.01^aA^
FCM-QE1	0.74 ± 0.01^dC^	0.81 ± 0.05^dB^	0.89 ± 0.05^A^
FCM-QE2	0.76 ± 0.02^cdC^	0.85 ± 0.04^cdB^	0.95 ± 0.01^abA^
FCM-QE3	0.79 ± 0.02^cB^	0.81 ± 0.02^dB^	0.96 ± 0.02^abA^

^a, b, c, and d^: A significant difference (*P* ≤ 0.05) exists between any two means within the same column with the different superscript letters. ^A, B, and C^: A significant difference (*P* ≤ 0.05) exists between any two means within the same row with the different superscript letters. FCM-C (control without quinoa), FCM-QS1 (1% quinoa sprout flour), FCM-QS2 (2% quinoa sprout flour), FCM-QS3 (3% quinoa sprout flour), FCM-QE1 (1% freeze-dried aqueous Quinoa extract), FCM-QE2 (2% freeze-dried aqueous Quinoa extract), and FCM-QE3 (3% freeze-dried aqueous Quinoa extract).

As a normal inverse relation, the greater the decrease in pH values, the greater the increase in the TA% was noted for all FCM samples during the storage period (15 days/4 °C ± 1). The more the storage period increased, the more the TA% increased in all FCM samples (Table 1). The TA% indicated a highly significant (p<0.05) difference in all produced FCM samples after 15 days of cold storage compared to their values after 1 or 7 days of storage for each sample. The supplementation of CM with quinoa sprout flour revealed a more significant increase in acidity when compared to the FCM-C sample and samples supplemented with different amounts of freeze-dried aqueous quinoa extract (1%: FCM-QE1, 2%: FCM-QE2, and 3%: FCM-QE3). The supplementation of CM with 3% quinoa sprout flour (FCM-QS3) or freeze-dried aqueous quinoa extract (FCM-QE3) resulted in a slight or no significant increase in TA%.

### 3.3 Chemical composition of different FCM samples

The chemical composition of standardized CM used in the current study and different FCM samples is presented in Table 2. The composition of the control FCM sample (FCM-C) was characterized by a non-significant change (*p* > 0.05) in all components when compared with the modified CM sample, except for TS and ash%, which significantly increased. The effect of adding quinoa sprout flour (1, 2, and 3%) and freeze-dried aqueous quinoa extract (1, 2, and 3%) on the chemical composition of FCM samples is also presented (Table 2). The tabulated data revealed that adding both quinoa preparations at all amounts of addition significantly decreased moisture and increased TS compared to the FCM-C sample. Regarding the effect of quinoa sprout flour, it is observed that there were no apparent changes between treatments (FCM-QS1, FCM-QS2, and FCM-QS3) in all components as affected by the amount added to CM except for protein (4.55 ± 0.03%) and fat (4.60 ± 0.06%) as they significantly (p<0.05) increased when 3% quinoa sprouts flour is added (FCM-QS3). Moreover, adding freeze-dried aqueous quinoa extract did not significantly affect the moisture and TS% between all FCM samples containing quinoa preparations. The ash was markedly higher in the FCM-QE3 (1.30 ± 0.00%) sample than in all other FCM samples. At the same time, the total carbohydrates and proteins were significantly higher in FCM-QS3 and FCM-QE3 compared to other treatments, with no significant differences between them.

**Table 2:** Chemical composition of modified CM and different FCM samples (mean ± SE), *n* = 3.

Sample	Moisture%	TS%	Protein%	Fat%	Ash%	Total carbohydrates %
Standardized CM	88.21 ± 0.19^a^	11.79 ± 0.19d	4.15 ± 0.12^c^	4.17 ± 0.03^c^	0.83 ± 0.03^e^	2.64 ± 0.17^c^
FCM-C	87.53 ± 0.31^ab^	12.47 ± 0.31^c^	4.43 ± 0.09^bc^	4.23 ± 0.09^bc^	0.89 ± 0.00^d^	2.91 ± 0.41^bc^
FCM-QS1	86.74 ± 0.36^bc^	13.26 ± 0.36^bc^	4.03 ± 0.12^c^	4.30 ± 0.07^abc^	1.17 ± 0.01^c^	3.75 ± 0.3^ab^
FCM-QS2	86.15 ± 0.29^cd^	13.85 ± 0.29^ab^	4.37 ± 0.19^bc^	4.37 ± 0.22^abc^	1.21 ± 0.01^bc^	3.91 ± 0.70^ab^
FCM-QS3	85.65 ± 0.57^cd^	14.35 ± 0.57^ab^	4.55 ± 0.03^a^	4.60 ± 0.06^a^	1.23 ± 0.03^b^	3.97 ± 0.05^a^
FCM-QE1	86.20 ± 0.16^cd^	13.80 ± 0.16^ab^	4.40 ± 0.12^bc^	4.32 ± 0.1^abc^	1.20 ± 0.01^bc^	3.53 ± 0.04^bc^
FCM-QE2	85.65 ± 0.34^d^	14.35 ± 0.34^a^	4.75 ± 0.03^ab^	4.45 ± 0.08^abc^	1.22 ± 0.01^bc^	3.70 ± 0.40^ab^
FCM-QE3	85.91 ± 0.20^d^	14.09 ± 0.20^ab^	4.98 ± 0.19^a^	4.50 ± 0.10^ab^	1.30 ± 0.00^a^	3.89 ± 0.15^ab^

^a, b, c, d, and e^: Significant differences (*P* ≤ 0.05) exist between any two means within the same column with the different superscript letters. FCM-C (control without quinoa), FCM-QS1 (1% quinoa sprout flour), FCM-QS2 (2% quinoa sprout flour), FCM-QS3 (3% quinoa sprout flour), FCM-QE1 (1% freeze-dried AQE), FCM-QE2 (2% freeze-dried AQE), and FCM-QE3 (3% freeze-dried AQE). Total carbohydrates include available carbohydrates and fiber.

### 3.4 Water holding capacity % and apparent viscosity (cp)

The physicochemical characteristics and viscosity of different FCM samples are presented in Table 3. Among all FCM samples produced under the conditions of the current study, the lowest WHC% and apparent viscosity (cp) were detected for the control sample (FCM-C). Generally, supplementing FCM samples with freeze-dried aqueous quinoa extract increased WHC% more than quinoa sprout flour. The significantly (*p* < 0.05) higher ability to bind water (WHC%: 66.35 ± 0.30) was observed for FCM-QE3. As a result of the storage period, a decrease in water binding capacity was observed for all FCM samples. The apparent viscosity was also affected by the type and amount of supplementation of quinoa preparations, as well as the storage period of the FCM samples. The viscosity values gradually decreased with increasing storage period for all FCM samples. The decline in viscosity values in stored fermented milk products happens because yogurt is a gel/matrix of casein micelles with entrapped water. Among all FCM samples, the highest viscosity after 1 day and 15 days of cold storage (4 °C ± 1) was 878.20 ± 9.48cp and 407.40 ± 1.74cp, respectively, as recorded for the FCM-QE3 sample. According to these findings, it could be reported that increasing viscosity in produced FCM samples was noticeable when freeze-dried aqueous quinoa extract was added, especially at a higher level (2%) than in other FCM samples.

**Table 3:** WHC%, and apparent viscosity (cp) of different FCM samples during 15 days of cold storage (4 °C ± 1) as affected by quinoa preparations addition (mean ± SE), *n* = 3.

Sample	WHC%			Apparent viscosity (cp)		
	Storage period (days)			Storage period (days)		
	1	7	15	1	7	15
FCM-C	37.25 ± 0.14^gA^	35.24 ± 1.13^fB^	34.12 ± 0.65^gC^	183.77 ± 3.90^eA^	145.17 ± 0.78^eB^	109.03 ± 0.61^eC^
FCM-QS1	46.80 ± 0.49^fA^	45.07 ± 0.07^eB^	41.30 ± 0.36^fC^	238.97 ± 1.30^dA^	222.07 ± 1.40^dA^	177.17 ± 1.58^dB^
FCM-QS2	47.90 ± 0.46^dA^	47.27 ± 0.25^cB^	46.37 ± 0.31^dC^	133.17 ± 3.03^fB^	223.53 ± 1.10^dA^	108.00 ± 0.44^eB^
FCM-QS3	61.07 ± 0.52^bA^	60.19 ± 0.21^aB^	51.68 ± 0.33^bC^	459.8 ± 5.70^cA^	479.20 ± 5.17^cA^	372.87 ± 0.78^bB^
FCM-QE1	46.83 ± 0.27^eA^	46.07 ± 0.11^dB^	44.33 ± 0.28^eC^	680.17 ± 104.27^bA^	530.67 ± 7.93^bB^	238.53 ± 2.26^cC^
FCM-QE2	49.52 ± 0.34^cA^	49.31 ± 0.32^bA^	48.50 ± 0.36^cC^	218.66 ± 1.17^dA^	229.90 ± 0.74^dA^	241.57 ± 0.69^cA^
FCM-QE3	66.35 ± 0.30^aA^	59.83 ± 0.30^aB^	57.52 ± 0.32^aC^	878.20 ± 9.48^aB^	1225.37 ± 17.68^aA^	407.40 ± 1.74^aC^

^a, b, c, d, e, f, and g^: A significant difference (*P* ≤ 0.05) between any two means within the same column with different superscript letters. ^A, B, and C^: A significant difference (*P* ≤ 0.05) between any two means within the same row has different superscript letters for each parameter. FCM-C (control without quinoa), FCM-QS1 (1% quinoa sprout flour), FCM-QS2 (2% quinoa sprout flour), FCM-QS3 (3% quinoa sprout flour), FCM-QE1 (1% freeze-dried AQE), FCM-QE2 (2% freeze-dried AQE), and FCM-QE3 (3% freeze-dried AQE).

### 3.5 Microbiological properties of different FCM samples

**Figure 5:**
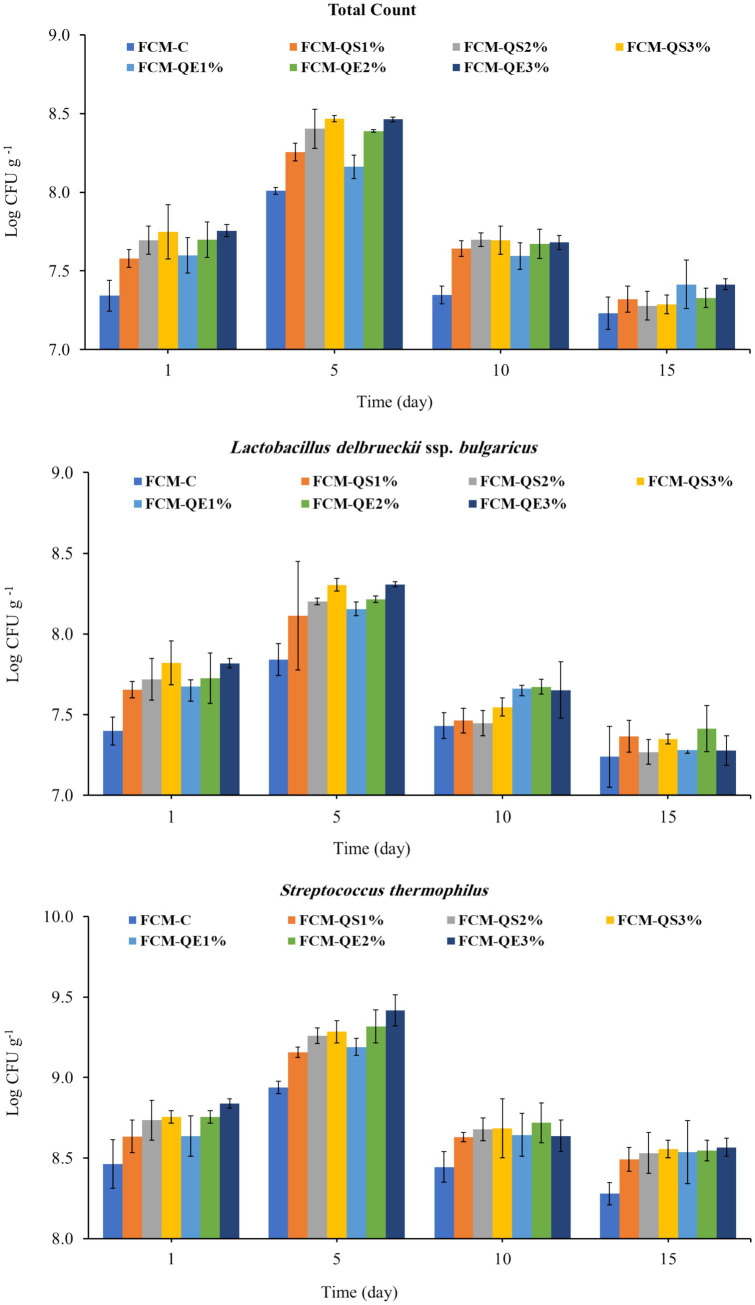
Viable bacterial groups (Log CFUg^−1^) of different FCM samples during 15 days of cold storage (4 °C ± 1). FCM-C (control without quinoa), FCM-QS1% (1% quinoa sprout flour), FCM-QS2% (2% quinoa sprout flour), FCM-QS3% (3% quinoa sprout flour), FCM-QE1% (1% freeze-dried AQE), FCM-QE2% (2% freeze-dried AQE), and FCM-QE3% (3% freeze-dried AQE) (mean ± SE), *n* = 3.

Regarding the microbiological quality of the prepared different FCM samples, no viability was detected for coliform, mold, or yeast groups. The viable bacteria enumerated on TC, MRS, and M17 media were counted, and their colony counts are presented in Figure 5. For all three media used in the study, it was observed that the FCM-QE3 sample had the highest viable bacterial counts. In contrast, the lowest counts were detected in the control sample (FCM-C). Moreover, the total bacterial counts, *Lactobacilli* sp. counts, and *Streptococcus thermophilus* counts peaked after 5 days of storage and showed the highest survival in each sample. The decline in viability happened gradually from day 10 to day 15 for all the examined samples in the current study. The difference in TC, *Streptococcus thermophilus*, and Lactobacilli counts between the treatments was significant (*p* < 0.05). The viability of all starter cultures used in the fermentation was increased by the addition of quinoa sprout flour or freeze-dried aqueous quinoa extract to 3% in FCM-QS3 and FCM-QE3, respectively, when compared to lower supplement levels (1 and 2%).

### 3.6 Antioxidant characteristics of different FCM samples

To evaluate the antioxidant properties of the different FCM samples as affected by type and amount of quinoa preparation, the total radical scavenging activity expressed by the DPPH (μmol TE g^−1^) method, TPC (mg GAE g^−1^), TFL (mg QE g^−1^), and TF (mg QE g^−1^) were determined, and the collected data are presented in Table 4. Significant differences between FCM samples were observed, and they were affected by the type and amount of the added quinoa preparation. Furthermore, the storage period presented an effect, Table 4. The more the quinoa preparation supplementation and the storage period increased, the more the total antioxidant activity was detected. The total antioxidant activity in μmol TE g^−1^ of the FCM-C sample free of either sprouted quinoa flour or freeze-dried aqueous quinoa extract was 10.83 and 31.36 on the first day of storage and after 15 days, respectively. After 1 day of storage, the FCM-C sample’s TPC was not detected, while FCM-QE3 showed a TPC of 60.91 mg GAE g^−1^ after 15 days of storage. When TFL and TF were not detected in the FCM-C sample, their values were significantly (*p* < 0.05) increased by increasing the quinoa preparation addition. The highest values of TFL and TF were 8.63 and 9.20 mg QE g^−1^, respectively, for the FCM-QE3 sample after 15 days of cold storage (4 ± 1 °C).

**Table 4:** Antioxidant properties of different FCM samples during 15 days of cold storage (4 °C ± 1) as affected by quinoa preparations addition (mean ± SE), *n* = 3.

Parameter	Storage period (days)	Sample						
		FCM-C	FCM-QS1	FCM-QS2	FCM-QS3	FCM-QE1	FCM-QE2	FCM-QE3
DPPH (μmol TE g^−1^)	1	10.83 ± 3.52^cB^	23.02 ± 6.59^bcB^	32.32 ± 10.54^abB^	44.50 ± 3.48^aB^	36.79 ± 11.36^abB^	46.12 ± 3.73^aB^	49.83 ± 0.17^aB^
	7	24.91 ± 8.75^cA^	27.01 ± 4.57^cB^	34.60 ± 4.48^bcB^	52.30 ± 12.72^aAB^	43.54 ± 4.58^abB^	49.12 ± 7.23^abB^	58.60 ± 17.33^aB^
	15	31.36 ± 4.65^eA^	41.33 ± 2.42^deA^	47.49 ± 8.23^cdA^	62.19 ± 17.41^cA^	62.15 ± 10.41^cA^	76.86 ± 4.83^bA^	110.63 ± 19.63^aA^
TPC (mg GAE g^−1^)	1	ND	47.50 ± 6.52^cdB^	49.26 ± 11.51^bcdB^	53.53 ± 1.41^abcB^	49.26 ± 11.51^bcdB^	55.63 ± 1.27^abA^	58.69 ± 0.82^aA^
	7	ND	53.85 ± 3.28^aA^	54.94 ± 0.61^aA^	56.16 ± 1.57^aAB^	53.47 ± 0.06^aB^	59.61 ± 0.61^aA^	59.98 ± 0.49^aA^
	15	ND	55.35 ± 1.27^aA^	58.09 ± 2.24^aA^	60.91 ± 0.43^aA^	59.47 ± 0.49^aA^	59.97 ± 0.40^aA^	60.91 ± 0.43^aA^
TFL (mg QE g^−1^)	1	ND	0.78 ± 0.21^deB^	0.49 ± 0.22^deC^	2.07 ± 0.35^cdB^	2.99 ± 0.16^cB^	5.94 ± 1.56^bB^	7.79 ± 0.39^aB^
	7	ND	0.92 ± 0.24^deB^	1.60 ± 1.04^cdB^	5.36 ± 1.08^bA^	2.61 ± 0.59^cB^	6.82 ± 0.01^aB^	7.87 ± 0.51^aB^
	15	ND	1.76 ± 0.09^dA^	6.05 ± 0.56^bA^	2.09 ± 0.30^dB^	4.59 ± 2.58^cA^	8.12 ± 1.40^aA^	8.63 ± 0.28^aA^
TF (mg QE g^−1^)	1	ND	0.73 ± 0.18^dB^	1.09 ± 0.24^dA^	2.05 ± 0.16^cB^	2.22 ± 0.20^cB^	5.65 ± 0.36^bA^	8.26 ± 0.06^aB^
	7	ND	0.82 ± 0.25^dB^	1.33 ± 0.18^dA^	2.97 ± 0.40^cA^	2.90 ± 0.49^cA^	4.78 ± 0.13^bB^	9.12 ± 0.44^aA^
	15	ND	3.15 ± 1.14^dA^	1.37 ± 0.25^eA^	3.01 ± 0.19^cA^	3.01 ± 0.37^cA^	6.19 ± 0.41^bA^	9.20 ± 0.55^aA^

ND: not detected. ^a, b, c, d, and e^: A significant difference (*P* ≤ 0.05) between any two means within the same row has different superscript letters; ^A and B^: A significant difference (*P* ≤ 0.05) between any two means within the column has different superscript letters for each parameter. FCM-C (control without quinoa), FCM-QS1 (1% quinoa sprout flour), FCM-QS2 (2% quinoa sprout flour), FCM-QS3 (3% quinoa sprout flour), FCM-QE1 (1% freeze-dried aqueous Quinoa extract), FCM-QE2 (2% freeze-dried aqueous Quinoa extract), and FCM-QE3 (3% freeze-dried aqueous Quinoa extract).

Generally, the effect of the cold storage period on the antioxidant parameters for all FCM samples was slightly significant under the conditions of the current study. After 1 day of storage, the FCM-QE3 showed a TPC of 60.91 mg GAE g^−1^ after 15 days of storage. When TFL and TF were not detected in the FCM-C sample, their values were significantly (*p* < 0.05) increased by increasing the quinoa preparation addition. The highest values of TFL and TF were 8.63 and 9.20 mg QE g^−1^, respectively, for the FCM-QE3 sample after 15 days of cold storage (4 ± 1 °C). Generally, the effect of the cold storage period on the antioxidant parameters for all FCM samples was slightly significant under the conditions of the current study.

### 3.7 Microstructure examination

**Figure 6:**
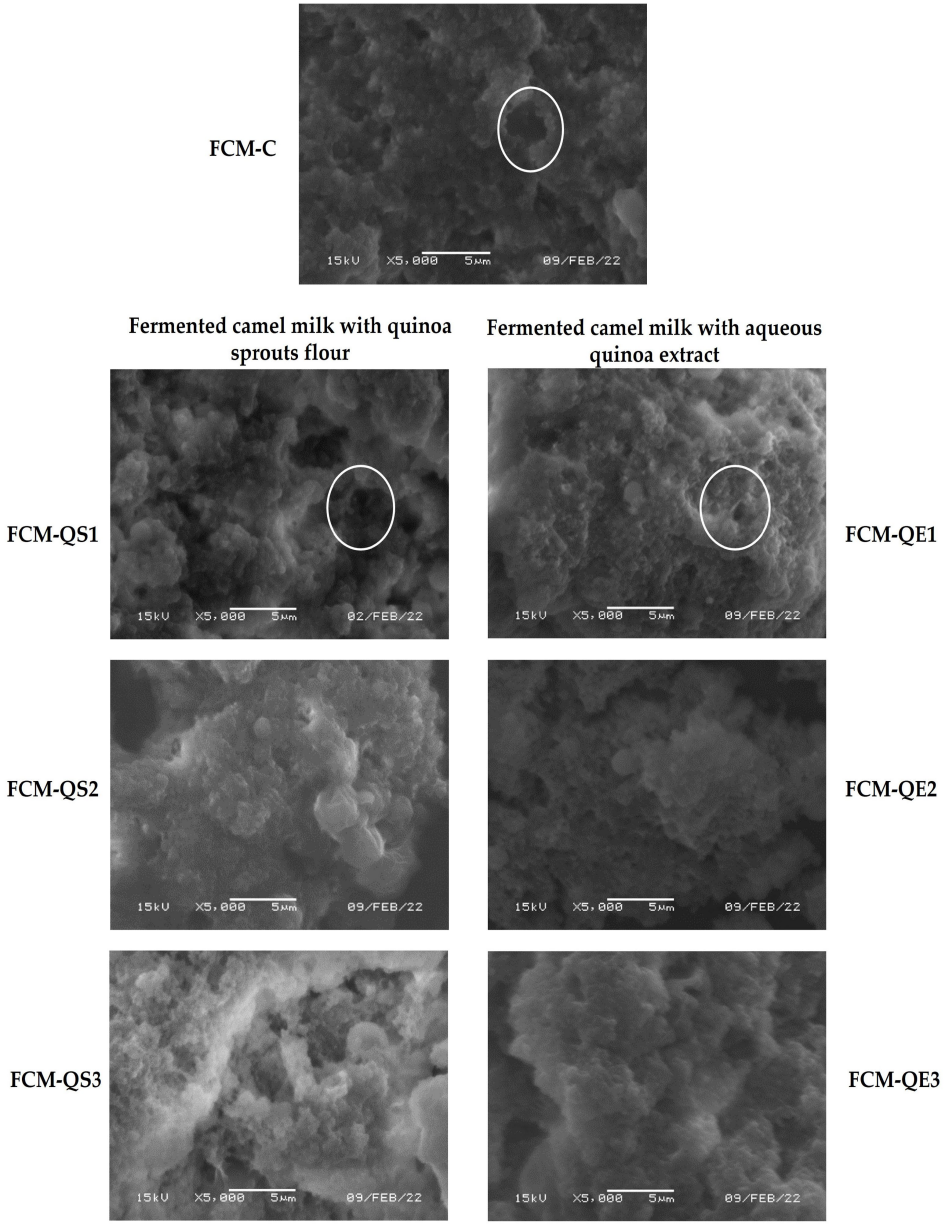
Scanning electron micrographs (×5,000) of different FCM samples. FCM-C (control without quinoa), FCM-QS1 (1% quinoa sprout flour), FCM-QS2 (2% quinoa sprout flour), FCM-QS3 (3% quinoa sprout flour), FCM-QE1 (1% freeze-dried aqueous quinoa extract), FCM-QE2 (2% freeze-dried aqueous Quinoa extract), and FCM-QE3 (3% freeze-dried aqueous Quinoa extract).

**Figure 7:**
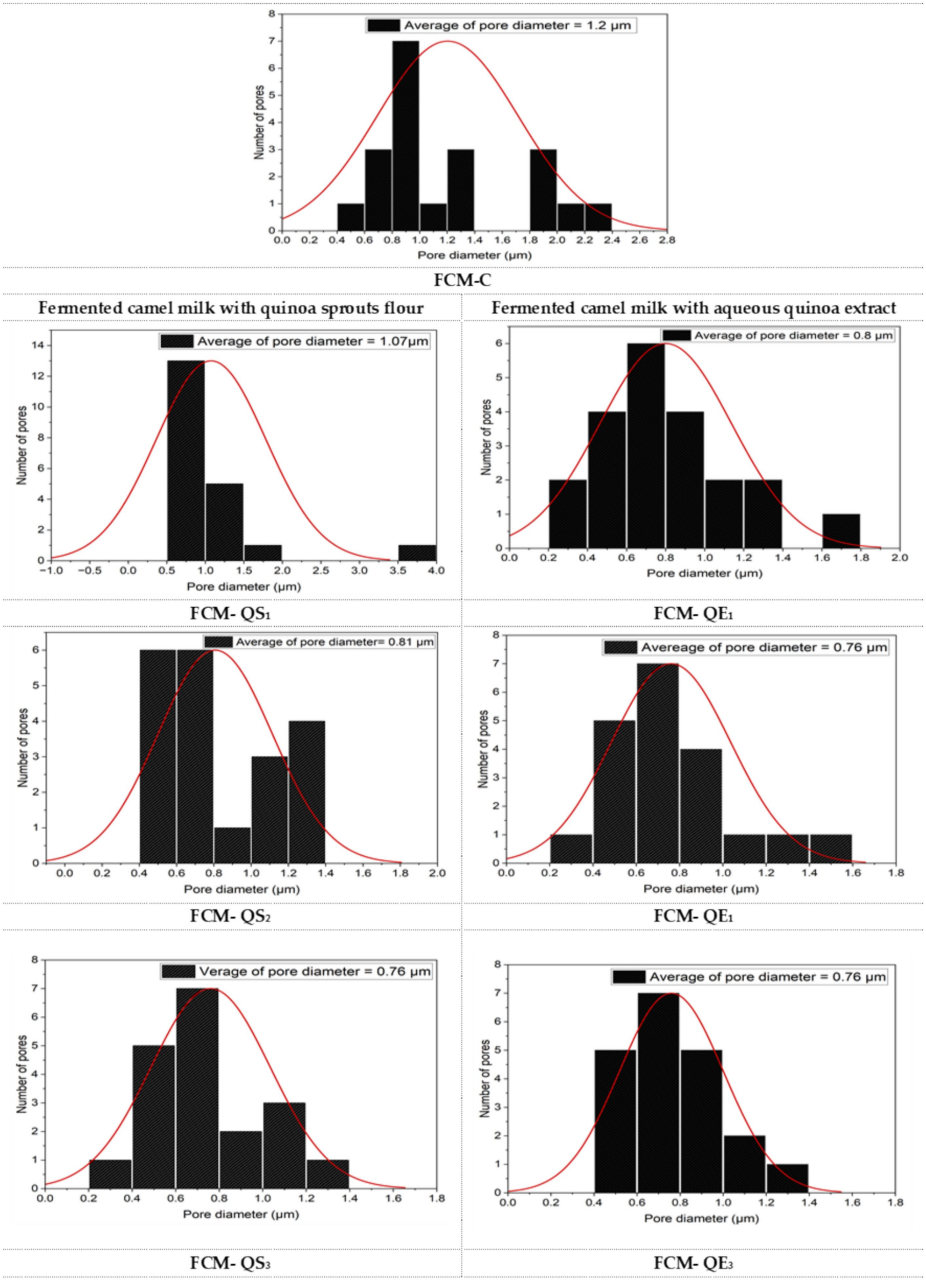
Average pore diameter (μm) appeared in the scanning electron micrographs of different FCM samples as calculated using ImageJ and presented by Origin 2023 software. FCM-C (control without quinoa), FCM-QS1 (1% quinoa sprout flour), FCM-QS2 (2% quinoa sprout flour), FCM-QS3 (3% quinoa sprout flour), FCM-QE1 (1% freeze-dried aqueous Quinoa extract), FCM-QE2 (2% freeze-dried aqueous Quinoa extract), and FCM-QE3 (3% freeze-dried aqueous Quinoa extract).

SEM examined the microstructure of different FCM samples. The electron micrographs at ×5,000 magnification for all samples are presented in Figure 6. For comparing protein matrix compactness and void spaces (white circumference circle), the average pore size diameter (μm) distributed in each micrograph’s field was calculated by Image J and presented by Origin 2023 software (Figure 7).

The SEM showed differences in the control (FCM-C) microstructure and other FCM samples. The differences were mainly associated with the compactness of the protein matrix and the reduction in the number and size of the void spaces containing the aqueous portion of the gel, which appeared by increasing the amounts of sprouted quinoa flour or freeze-dried aqueous quinoa extract. Moreover, the calculated average pore diameter (μm) that appeared in the scanning electron micrographs of different FCM samples is presented in Figure 7.

As can be seen, the average pore diameter in the control fermented CM sample was the highest (FCM-C: 1.2 μm). The pore size diameter in samples containing sprouted quinoa flour was 1.07, 0.81, and 0.76 μm for FCM-QS1, FCM-QS2_,_ and FCM-QS3, respectively. A higher decrease in porosity was found by adding freeze-dried aqueous quinoa extract compared to other samples. The pore size diameters for FCM-QE1, FCM-QE2, and FCM-QE3 were 0.80, 0.76, and 0.076 μm, respectively.

### 3.8 Instrumental color analysis

The measured color parameters of lightness (L*), redness (a*), and yellowness (b*) and the calculations of chroma (C), color changes (∆E), and browning index (BI) for different FCM samples contained various amounts of quinoa preparations compared to their values in the control sample (FCM-C) during storage are presented in Table 5. The L* values increased significantly as the storage time increased for all samples. The sample supplemented with freeze-dried aqueous quinoa extract revealed a significant increase in L* values compared to sprouted quinoa flour-supplemented samples. In contrast, it was observed that the values related to yellowness (b*) increased as the storage period and/or level of quinoa preparation supplementation increased. Obviously, it could be seen that the effect of the sprouted quinoa flour addition on turning the color towards yellowness was more significant than the freeze-dried aqueous quinoa extract addition. Moreover, the colorfulness of the samples appeared more evident by increasing the storage period, which was significantly higher for the FCM-QS3 (8.16) sample than the FCM-QE3 (6.80) sample. So, it could be said that an adverse effect on the bright white color of the fermented CM happened with the addition of sprouted quinoa flour when compared to the freeze-dried aqueous quinoa extract addition under the conditions of the current study.

**Table 5:** Instrumental color measurements of different FCM samples during 15 days of cold storage (4 °C ± 1) as affected by the addition of quinoa preparations addition.

Color parameter	Storage period (day)	Sample						
		FCM-C	FCM-QS1	FCM-QS2	FCM-QS3	FCM-QE1	FCM-QE2	FCM-QE3
L*	1	86.97 ± 0.00^aC^	86.58 ± 0.00^bB^	86.14 ± 0.01^cB^	84.82 ± 0.02^gC^	85.54 ± 0.00^dC^	85.26 ± 0.01^eC^	85.04 ± 0.04^fC^
	7	87.99 ± 0.02^cA^	86.23 ± 0.00^eC^	88.89 ± 0.01^aA^	86.08 ± 0.00^gA^	88.38 ± 0.01^bB^	86.25 ± 0.00^fB^	87.25 ± 0.02^dB^
	15	87.69 ± 0.02^cB^	88.72 ± 0.01^bA^	84.31 ± 0.05^eC^	84.90 ± 0.01^dB^	88.82 ± 0.02^aA^	87.68 ± 0.06^cA^	87.65 ± 0.08^cA^
a*	1	−1.73 ± 0.00^aA^	−1.87 ± 0.00^bcA^	−1.92 ± 0.01^cdB^	−1.80 ± 0.00^bA^	−1.89 ± 0.00^cA^	−1.99 ± 0.01^dB^	−2.18 ± 0.00^eA^
	7	−1.73 ± 0.01^aA^	−1.85 ± 0.01^bA^	−1.69 ± 0.01^aA^	−1.76 ± 0.01^aA^	−2.09 ± 0.03^cB^	−1.89 ± 0.00^bA^	−2.51 ± 0.04^dB^
	15	−1.90 ± 0.03^aB^	−2.12 ± 0.01^bB^	−2.35 ± 0.04^cC^	−2.19 ± 0.02^bB^	−2.45 ± 0.02^dC^	−2.49 ± 0.11^dC^	−2.60 ± 0.13^eC^
b*	1	2.37 ± 0.00^gB^	4.90 ± 0.00^cB^	5.33 ± 0.02^bB^	7.92 ± 0.02^aB^	2.79 ± 0.01^fC^	3.02 ± 0.01^eB^	3.99 ± 0.04^dC^
	7	2.36 ± 0.01^fB^	3.42 ± 0.01^dC^	3.22 ± 0.00^eC^	8.04 ± 0.00^aA^	4.28 ± 0.01^cB^	2.39 ± 0.00^fC^	6.08 ± 0.02^bB^
	15	2.57 ± 0.02^gA^	5.71 ± 0.00^dA^	6.74 ± 0.11^bA^	7.86 ± 0.00^aC^	4.91 ± 0.02^eA^	4.56 ± 0.08^fA^	6.28 ± 0.10^cA^
C	1	2.94 ± 0.00^gB^	5.25 ± 0.01^cB^	5.67 ± 0.02^bB^	8.12 ± 0.02^aB^	3.37 ± 0.01^fC^	3.62 ± 0.02^eB^	4.54 ± 0.04^dC^
	7	2.92 ± 0.01^gB^	3.89 ± 0.01^dC^	3.64 ± 0.01^eC^	8.23 ± 0.00^aA^	4.76 ± 0.01^cB^	3.05 ± 0.00^fC^	6.58 ± 0.04^bB^
	15	3.19 ± 0.03^gA^	6.09 ± 0.00^dA^	7.14 ± 0.12^bA^	8.16 ± 0.01^aB^	5.49 ± 0.03^eA^	5.20 ± 0.12^fA^	6.80 ± 0.14^cA^
Bl	1	1.25 ± 0.01^gB^	4.10 ± 0.00^cB^	4.60 ± 0.03^bB^	8.00 ± 0.03^aB^	1.63 ± 0.02^fC^	1.82 ± 0.01^eB^	2.80 ± 0.04^dC^
	7	1.22 ± 0.01^fB^	2.38 ± 0.01^dC^	2.22 ± 0.00^eC^	8.05 ± 0.01^aA^	3.11 ± 0.02^cB^	1.15 ± 0.00^gC^	4.93 ± 0.01^bB^
	15	1.32 ± 0.01^gA^	4.73 ± 0.00^dA^	6.07 ± 0.12^bA^	7.57 ± 0.01^aC^	3.52 ± 0.01^eA^	3.13 ± 0.01^fA^	5.06 ± 0.01^cA^
ΔE	1	ND	2.57 ± 0.01^cB^	3.09 ± 0.02^bB^	5.96 ± 0.01^aA^	1.51 ± 0.00^eC^	1.85 ± 0.02^dB^	2.56 ± 0.01^cC^
	7	ND	1.30 ± 0.00^eC^	2.10 ± 0.01^dC^	5.74 ± 0.00aC	2.39 ± 0.01 ^c B^	0.75 ± 0.00^fC^	3.80 ± 0.03^bB^
	15	ND	3.79 ± 0.01^dA^	5.16 ± 0.12^bA^	5.89 ± 0.01^aB^	3.21 ± 0.03^eA^	2.42 ± 0.12^fA^	4.06 ± 0.13^cA^

^a, b, c, d, e, f, and g^: A significant difference (*P* ≤ 0.05) between any two means within the same row has a different superscript letter. ^A, B, and C^: A significant difference (*P* ≤ 0.05) between any two means within the column has a different superscript letter for each parameter. FCM-C (control without quinoa), FCM-QS1 (1% quinoa sprout flour), FCM-QS2 (2% quinoa sprout flour), FCM-QS3 (3% quinoa sprout flour), FCM-QE1 (1% freeze-dried aqueous Quinoa extract), FCM-QE2 (2% freeze-dried aqueous Quinoa extract), and FCM-QE3 (freeze-dried aqueous Quinoa extract).

## 4 Discussion

The results of the current study indicated that combining camel milk with WPI and TSC, along with the addition of freeze-dried aqueous quinoa extract, significantly improved the fermentation behavior, structural integrity, and antioxidant properties of yogurt-like products. This approach presents a promising strategy for addressing the inherent technological challenges associated with using camel milk in fermented dairy products.

According to Mohamed et al. (45), manufacturing fermented dairy products like yogurt from CM is difficult because the acid curd produced from CM is fragile and has a thin consistency and soft texture caused by dispersed flakes and heterogeneity. The lack of *β*-lactoglobulin and the predominance of *α*-lactalbumin in the CM whey proteins may be the main reason behind the appearance of the fragile structure of fermented CM products. Moreover, the difference in casein composition and micellar size between camel and bovine milk is responsible for its weak gelation properties when used to make yogurt and cheese. Attia et al. (46) explained that CM’s larger micelle size can be attributed to its higher mineral content than bovine milk. Camel casein micelles contain higher levels of citrate salts (~98 mg g^−1^) than their content in bovine micelles (67 mg g^−1^). Magnesium, phosphorus, and citrate proportions in camel casein micelles are also reported to be higher in CM than in bovine milk. These results could be supported by the action of calcium chelating (i.e., TSC) that alters the technological properties of casein, as previously discussed by Broyard and Gaucheron (47). They reported that removing milk’s buffering capacity happens when calcium chelators are added.

In some cases, the buffering capacity increases because the chelating molecules have acid-basic groups that can bind protons and resist the decrease in pH. However, the maximal buffering capacity is shifted toward a lower or higher pH depending on the added chelating’s specific stability constant (pK values). In parallel to our findings, the study by Abou-Soliman et al. (3) aimed to investigate the effect of CM fortification with 1 and 2% skimmed milk powder (SMP), 1 and 2% WPC, or 0.4 and 0.5% *β*-lactoglobulin (β-Lg) on different characteristics of camel-milk yogurt coagulated by the ordinary yogurt starter culture with the impact of MTGase during fermentation. They found that the best concentration of dairy ingredients was 2, 2, and 0.5% for SMP, WPC, and β-Lg, respectively. Moreover, they reported that the fortification of CM with dairy powder ingredients such as SMP, WPC, or β-Lg markedly reduced the fermentation time of CM to a pH of about 4.6. According to their result, the reduction in fermentation time was recorded at approximately 25 min. Only β-Lg (0.5%) was added to CM compared with unfortified CM treatment. They concluded that although the fortification of CM with dairy powder ingredients such as SMP, WPC, or β-Lg did not improve the texture of CM yogurt, it positively affected fermentation time, as the time needed for pH to reach about 4.6 was decreased. Furthermore, these findings were similar to those previously reported by Sakandar et al. (48). They evaluated the effects of adding different concentrations (0, 2, 4, 6, and 8%) of polymerized WPI solution (10%) as a thickening agent on the properties of CM stirred yogurt. They showed that replacing CM with about 8% polymerized WPI improved the characteristics of stirred yogurt from CM and slightly affected pH and acidity. Chen et al. (11) also observed the positive effect of TSC on improving the viscosity and structure of CM acid gel. They found that the acid gel structures were enhanced as the TSC concentration increased from 0 to 30 mmol L^−1^. They explained the role of TSC in improving the acid gel properties of CM by dissociating casein micelles. TSC caused the casein micelles in CM to be dissociated into smaller casein particles by chelating the calcium ions of colloidal calcium phosphate. This resulted in more stable crosslinked casein particles, causing the acid gel to improve. Earlier, Ozcan-Yilsay et al. (16) indicated that adding TSC (between 10 and 15 mM) to milk improved the textural properties of acid gel and reduced the whey separation.

Moreover, the calcium chelates alter the technological properties of casein by forming small aggregates (30-50 nm), which leads to an increase in viscosity, as Broyard and Gaucheron (47) confirmed. From the obtained results, it could be noted that the co-fermentation of yogurt starter culture (YC-381) and *L. fermentum* (B-1392) of modified CM by the addition of WPI (2%) along with TSC (30 mmol L^−1^) can help accelerate acidity progression, improve acid coagulum, and increase milk viscosity. Thus, the addition of WPI (2%) and TSC (30 mmol L^−1^) was chosen to modify CM before fermentation in the rest of the study.

The results regarding pH and TA% findings were in parallel with those obtained by Codinǎ̓ et al. (21). They found that the supplementation of cow milk yogurt with quinoa flour up to 2% caused a much higher decrease in the pH value and a much higher increase in the total acidity value compared with the non-supplemented control sample. Regarding the effect of quinoa extract on the acidity development during milk fermentation, previous research focused on the impact of partially replacing goat milk either with water-soluble quinoa or permeate-soluble quinoa extracts at different levels (5–45 g 100 g^−1^) on the yogurt characteristics made by El-Shafei et al. (25). They indicated a higher rate of acid production and a higher decline in pH during the fermentation process compared to the control sample without quinoa extract milk replacement, which was also observed. As an explanation, this trend can be mainly due to the presence of quinoa proteins (49), the availability of amino acids (50), and the mineral content (51) of the quinoa flour necessary for the growth of the yogurt starter culture (21).

The results of WHC% were highly related to the increase in acidity. A similar trend was found by Alkobeisi et al. (24). They noted that casein network rearrangements due to increasing acidity during cold storage and the interaction between leached amylose and amylopectin chains increased syneresis levels during storage. In contrast to our results, it was reported by Codinǎ et al. (21) that more whey separation was measured in CM yogurt samples containing higher levels of quinoa. This is due to the flour being related to the unstable protein network, which can be induced by damage to the weak gel network. The opposite trend to our result may be due to the type of milk used in the study and the modification of CM proteins by adding WPI (2%) and TSC (30 mmol L^−1^).

Moreover, starch and fiber in quinoa flour could reduce free water molecules, mainly due to their water-binding ability (24). The addition of non-dairy substances may interrupt the gel structure of the enriched sample. Thus, the apparent viscosity of yogurt during storage time decreases or increases over time due to the rearrangement of protein and protein–protein contacts (52). Despite the increase in yogurt viscosity associated with the rise in quinoa flour content (53), the effect of freeze-dried aqueous quinoa extract was more apparent, as our results show. When quinoa flour was added at 1.5% to milk yogurt, the increase in viscosity was higher than in other samples with fewer levels of quinoa flour due to the high binding properties of quinoa flour and high starch granules rich in amylopectin used as a thickener in frozen and fermented foods (54). This could be due to the more incredible release of starch with the water-soluble portion while preparing the water quinoa extract, as El-Shafei et al. (25) mentioned. They found a significant statistical correlation between the increase in viscosity and replacing goat milk with water and quinoa extract. Moreover, they noted that in yogurt processing, heating milk to a temperature over the gelatinization temperature of quinoa starch (from 57 to 64 °C) before fermentation was sufficient for starch gelatinization to occur in milk, thus increasing the viscosity in the final product.

Previously, Casarotti et al. (55) stated that flour made from quinoa is high in carbohydrates, fiber, essential fatty acids, and microelements (potassium, phosphorus, magnesium, and calcium). Moreover, the significantly higher increase in the viable bacterial groups in FCM-QE3 than those counted in the FCM-QS3 reflected the greater availability of nutrients needed for bacterial strains when freeze-dried aqueous quinoa extract was added, compared to the addition of flour from sprouted quinoa. In parallel with our results, the findings of Akkoyun and Arslan (49) were reported. They found that at the beginning of storage, when the amount of quinoa flour in Aryan production was increased to 0.2%, the starter bacterial strain count was higher than that of samples without quinoa (56). Previously, Codină et al. (21) stated that adding quinoa flour up to 3 g 100 g^−1^ positively affected the development of starter yogurt bacteria due to decreased pH and increased total acidity. Starting from day 10 of the storage period, the opposite behavior was observed in the survival of different bacterial groups. This could be related to a significant increase in acidity and a decrease in pH values, as presented. As an explanation, Sadoud et al. (57) reported that lactic acid bacteria can remain active in fermented milk stored at 4 °C, but this negatively affects their viability, which depends on the culture type and carbon source. Furthermore, the viability of bacterial strains in fermented dairy products is affected by multiple factors such as acidity, pH, dissolved oxygen content, H_2_O_2_, storage temperature, bacterial culture strains, and lactic acid concentration (58). In the same trend, it was reported that it is more applicable for food applications to use only hot water as a green extraction technique, which helps increase the extraction efficiency and selectivity of antioxidant substances from quinoa (59). Moreover, Akkoyun and Arslan (49) noted that the total antioxidant activity in Ayran samples supplemented with quinoa flour was significantly increased on the 7th day and continued to increase on the 14th day for all samples. As previously reported, Quinoa seeds are an excellent source of various phenolic compounds. These compounds include a diverse array of phenolic acids, such as ferulic, vanillic, and protocatechuic acids, as well as flavonoids, particularly quercetin and kaempferol glycosides. The strong antioxidant and health-promoting properties of these compounds make quinoa a valuable ingredient in functional foods and nutrition (60, 61).

Moreover, the germination process can alter the polyphenols by increasing their contents and antioxidant properties (32). So, there is no doubt that the more quinoa preparations added to the FCM, the more the TPC, TFL, and TF values increased. Previously, the antioxidants of prepared yogurt-like beverages using lactic acid bacteria and quinoa flour were evaluated (62). In this study, the increase in total phenols and phenolic compounds detected after fermentation and during storage corresponded to a proportional increase in total antioxidant activity. The authors said that this phenomenon happened as a result of fermentation, according to their explanation, which occurred due to the combined effects of acidification, affecting solubility, and microbial hydrolytic enzymes that further promote the release of free phenolic compounds.

In line with our findings, the results of Ho et al. (63) regarding the microstructure of native CM acid gel network explained that CM gels had a similar structure with a coarse protein network and a few linked protein aggregates. Furthermore, the study by El-Shafei et al. (25) can support our results. Replacing goat milk with liquid aqueous extract from white quinoa seeds by 5, 10, and 15% increased the starch/total solids ratio. They reported, therefore, that goat yogurts with quinoa extracts exhibited more compacted microstructure than control goat yogurts after examining their samples by transmission electron microscope (TEM). In the casein particle network, the starch appeared surrounded by aggregated protein particles and occupied the void space with varying dimensions. The explanation reported by Alkobeisi et al. (24) agreed with ours. Furthermore, they found that when skimmed milk powder was replaced with 100% quinoa flour in concentrated yogurt, a more compact and stronger gel structure of the gel network was found, and they revealed that the increase in carbohydrates in the formula is a reason. They also stated that proteins can form bonds with starch molecules through hydrophilic groups. Starch acts as a filler compound within the protein matrix, increasing gel strength and covering some areas of casein clusters and network pores.

Moreover, Codinǎ̓ et al. (21) reported that when quinoa flour was added to yogurt milk up to 1.0%, the lowest value of whey loss and good porosity of protein network were found. In general speech, it could be said that adding freeze-dried aqueous quinoa extracts positively affected the acid gel network from CM pre-modified by the addition of TSC and WPI. Thus, the microstructure images obtained complied with the increasing WHC and decreasing syneresis obtained, showing that a high level of freeze-dried aqueous quinoa extract addition reduced the diameter of interspaces, thus leading to a more stable gel network during cold storage following the whey separation obtained.

Regarding the instrumental color analysis, the same finding was also reported by Alkobeisi et al. (24). They found that replacing skimmed milk powder with quinoa flour up to 100% in concentrated yogurt caused a significant reduction in L* values compared to the control sample free of quinoa flour. Thus, the degree of brightness. Although a* value for all samples was negative, it decreased in all FCM samples as storage time increased. This is probably due to the increased whey separation during storage, which contains riboflavin (64).

## 5 Conclusion

In conclusion, standardized CM with WPI (2%) and TSC (30 mmol L^−1^) markedly enhanced fermentation characteristics. This alteration facilitated the acceleration of acidity development, improved coagulation, and augmented viscosity. The study investigated the creation of fermented CM samples by incorporating sprouted quinoa flour or freeze-dried aqueous quinoa extract at concentrations of 1, 2, or 3% into the fortified milk. The findings demonstrated that elevated quinoa concentrations increased acidity and enhanced water-holding capacity in fermented CM. The viable bacterial counts of the starter cultures increased by 3%, with both quinoa preparations exerting a greater influence on freeze-dried aqueous quinoa extract than on sprouted quinoa flour. Moreover, the antioxidant capabilities of the milk are enhanced with increased supplementation amounts. A microstructural study demonstrated that samples treated with 3% freeze-dried aqueous quinoa extract exhibited greater compactness and reduced pore size. The sensory evaluation indicated that the texture of fermented CM products enhanced with quinoa preparations improved. A slight decrease in flavor was observed, leading to diminished total sensory scores for all quinoa-supplemented samples relative to the control. It is advisable to incorporate quinoa preparations to enhance CM’s texture, microstructure, and antioxidant qualities. Future investigations should aim to optimize the critical processing parameters involved in camel milk fermentation. The incorporation of functional ingredients and the application of advanced fermentation technologies are expected to enhance the nutritional profile, physicochemical characteristics, and sensory quality of the resultant products. Moreover, systematic evaluation of consumer acceptance will be essential for the successful commercialization of fermented camel milk. In parallel, exploring alternative preparations derived from quinoa seeds represents a promising research direction, as their inclusion may further improve the texture, sensory attributes, and antioxidant potential of fermented camel milk formulations.

## Data Availability

The original contributions presented in the study are included in the article/supplementary material, further inquiries can be directed to the corresponding author.
